# *Rbm14* maintains the integrity of genomic DNA during early mouse embryogenesis via mediating alternative splicing

**DOI:** 10.1111/cpr.12724

**Published:** 2019-12-03

**Authors:** Jing Li, Chenxin Wang, Guihai Feng, Linlin Zhang, Guilai Chen, Hao Sun, Jiaqiang Wang, Ying Zhang, Qi Zhou, Wei Li

**Affiliations:** ^1^ School of Life Sciences University of Science and Technology of China Hefei China; ^2^ State Key Laboratory of Stem Cell and Reproductive Biology Institute of Zoology Chinese Academy of Sciences Beijing China; ^3^ Institute for Stem Cell and Regeneration Chinese Academy of Sciences Beijing China; ^4^ University of Chinese Academy of Sciences Beijing China; ^5^ College of Life Science Northeast Agricultural University of China Harbin China

## Abstract

**Objective:**

In this study, we generated an *Rbm14* knockout mouse model to explore its functions during early mouse embryogenesis.

**Materials and methods:**

The *Rbm14* knockout mouse model was generated by a combination of clustered regularly interspaced short palindromic repeats (CRISPR)/Cas9 and microinjection techniques. The developmental defects of the knockout embryos were characterized by histological analyses. The accumulation of DNA damage in mouse embryonic stem cells (ESCs) was detected by γH2AX staining and comet assay. The altered mRNA splicing of DNA damage response (DDR)‐related genes was detected by RNA‐Seq analysis and confirmed by semi‐quantitative PCR. The interaction of RBM14 with alternative splicing‐related genes was detected by immunoprecipitation‐mass spectra (IP‐MS) and confirmed by co‐immunoprecipitation (Co‐IP).

**Results:**

*Rbm14* knockout in mice results in apoptosis and cell proliferation defects in early post‐implantation epiblast cells, leading to gastrulation disruption and embryonic lethality. FACS and immunostaining demonstrate accumulation of DNA damage in *Rbm14* knockout ES cells. We also identified altered splicing of DDR‐related genes in the knockout mouse ESCs by RNA‐Seq, indicating that RBM14‐mediated alternative splicing is required for the maintenance of genome integrity during early mouse embryogenesis.

**Conclusions:**

Our work reveals that *Rbm14* plays an essential role in the maintenance of genome integrity during early mouse embryonic development by regulating alternative splicing of DDR‐related genes.

## 1 INTRODUCTION

RNA binding proteins (RBPs) are a diverse protein family that is designated by their ability to bind to single or double strand RNAs. RNA transcripts are recognized and covered by RBPs as soon as they are synthetized and form ribonucleoprotein (RNP) complexes.^1^, ^2^ RBPs are reported to be involved in various RNA metabolic processes, including transcription,^3^ editing,^4^ splicing,^5^, ^6^ transport^7^ and translation.^8^ RBPs recognize RNA through specific amino acid sequences, such as the RNA recognition motif (RRM), arginine‐rich motif (ARM), K homology domain (KHD) and arginine‐glycine‐glycine (RGG) box.^9^ As one of the largest subgroups of single strand RNA binding proteins in eukaryotes,^10^ RRM family proteins are reported to be involved in multiple cellular functions and diseases, such as germ cell development,^11^ senescence^12^ and malignancy.^13^

Many RRM proteins have been reported to be implicated in mammalian embryonic development. For example, RNA binding motif protein 15 (RBM15), also known as OTT1, is highly expressed in various tissue types. The germ line deletion of *Rbm15* gene in mice results in defects in placental trophoblast development and placental vascular branching morphogenesis, thus leads to embryonic lethality beyond E9.5.^14^ While a conditional deletion of *Rbm15* within the hematopoietic compartment blocks B‐cell development.^15^ Another RRM family protein, RBM20, is a tissue‐specific pre‐mRNA splicing factor that is highly expressed in human heart. RBM20 mediates the alternative splicing of specific mRNA variants of many genes in cardiac muscles.^6^ Mutations in *Rbm20* gene are reported to be related with dilated cardiomyopathy in humans.^16^

RBM14 is an RBP that contains two RRMs in the N‐terminus and a prion‐like domain (PLD) in the C terminus.^17^ With its ability to interact with both RNAs and proteins, RBM14 acts as a multifunctional protein in eukaryotic cells and is reported to be implicated in many aspects of cellular processes such as transcription coactivation,^18^ alternative splicing,^19^ spindle assembly,^20^ DNA repair^21^ and cell differentiation.^22^ Recently, we had reported that RBM14 participates in pluripotency maintenance and mesoderm development of mouse embryonic stem cells (ESCs).^23^ However, the in vivo function of RBM14 in mammalian embryogenesis remains unclear. In this study, we investigated the role of RBM14 in mouse embryonic development using a *Rbm14* knockout mouse model generated through clustered regularly interspaced short palindromic repeats (CRISPR)/Cas9.^24^ Depletion of RBM14 causes DNA damage accumulation and cell proliferation defects, thus leads to the arrest of embryogenesis during gastrulation. Further studies demonstrate that RBM14 plays a vital role in the maintenance of genome integrity during early mouse embryogenesis through regulating alternative splicing of genes associated with DNA damage response (DDR).

## 2 MATERIALS AND METHODS

### 2.1 Animals

The ICR mice were purchased from the Beijing Vital River Laboratory Animal Center. All mice were housed under specific pathogen‐free (SPF) conditions in the animal facilities of the Institute of Zoology, Chinese Academy of Sciences. All animal experiments were approved by the Committee on Animal Care at the Institute of Zoology, Chinese Academy of Sciences. All institutional and national guidelines for the care and use of laboratory animals were followed.

### 2.2 Generation of the *Rbm14* knockout allele

The construction of the T7‐sgRNA plasmid was performed as described previously.^25^ The single stranded sgRNA oligonucleotides synthesized by the Beijing Genomics Institute (BGI) were annealed to form 5′ and 3′ overhangs, which are complementary to the sticky ends of *BbsI*‐digested T7‐gRNA backbone. The sequence of the oligonucleotides is listed in Table S1. The T7‐sgRNA and T7‐Cas9 plasmids were linearized using the endonuclease to serve as templates for in vitro transcription with the HiScribe T7 In Vitro Transcription Kit (New England Biolabs), following the manufacturer's instructions. The Cas9 mRNA and sgRNA were introduced into the fertilized eggs by microinjection. Each egg was injected with 2.5 ng of sgRNA and 10 ng of Cas9 mRNA. The injected embryos were cultured in vitro for 2 hours before implantation into the oviducts of the pseudopregnant female mice. Full‐term pups were obtained by natural labour 19.5 days post coitum (dpc). The genotype of the pups was determined using the tail tip DNA and primers F and R (sequences listed in Table S1). A Δ68 knockout allele that caused premature termination of RBM14 translation was identified in a male founder mouse.

### 2.3 Micromorphological observation

Early postimplantation embryos at E5.5, E6.5 and E7.5 stages obtained after interbreeding of *Rbm14* ^+/−^ male and female mice were dissected from the uteruses of the pregnant female mice. The size and morphology of the embryos were observed and imaged using a Leica orthographic microscope. The embryos were subjected to genotyping using primers F and R.

### 2.4 Haematoxylin and eosin staining

Haematoxylin and eosin (H&E) staining were performed as described previously.^25^ Briefly, E7.5 stage embryos obtained after interbreeding of *Rbm14* ^+/−^ male and female mice were dissected and fixed with 4% paraformaldehyde. The fixed embryos were dehydrated in an ethanol series (70%, 80%, 90% and 100% ethanol) and embedded in paraffin. The embryos were cut into 3 μm sections from the paraffin‐embedded blocks using a Leica slicing machine (Leica Biosystems) and mounted on poly‐d‐lysine‐coated glass slides (Zhong Shan Golding Bridge Biotechnology). The slides were heated at 65°C for 2 hours and immersed in xylene to remove the paraffin. After rehydration in an ethanol series (100%, 100%, 90%, 80% and 70% ethanol), the sections were stained with H&E following standard methods and imaged using a Leica Aperio VERSA 8 microscope (Leica Biosystems).

### 2.5 Immunofluorescent staining

Immunofluorescent staining was performed as described previously.^25^ For pre‐implantation embryos, the embryos obtained after interbreeding of *Rbm14* ^+/−^ male and female mice were flushed from the uteruses of the pregnant female mice and fixed with 4% paraformaldehyde. For ESCs, the cells were plated on 15‐mm round coverslips and cultured for one or two days and fixed with 4% paraformaldehyde. For postimplantation embryos, the embryos obtained after interbreeding of *Rbm14* ^+/−^ male and female mice were dissected from the uterus of the pregnant female mice, fixed, dehydrated and embedded in paraffin followed by sectioning. The sections were then dewaxed and rehydrated before staining. The fixed embryos, cells or rehydrated sections were subjected to immunostaining with appropriate primary antibodies and corresponding secondary antibodies following standard methods. The fluorescent images of the samples were captured using a Carl Zeiss LSM 780 confocal system. The primary antibodies and appropriate fluorophore (FITC, Cy3 and Cy5)‐conjugated secondary antibodies used in this study are listed in Table S2.

### 2.6 Bromodeoxyuridine (BrdU) labelling

Pregnant *Rbm14* ^+/−^ female mice (mated with *Rbm14* ^+/−^ male mice) were injected with BrdU (Sigma) intraperitoneally at a dose of 50 μg BrdU/g body weight. Mice were sacrificed 1 hour later, and the embryos were dissected for immunofluorescent staining.

### 2.7 Cell culture

Embryonic stem cell lines were isolated from the inner cell mass (ICM) of E3.5 blastula‐stage embryos obtained after interbreeding of *Rbm14* ^+/−^ male and female mice. The embryos were flushed from the uteruses of the pregnant female mice and plated on the dishes coated with fibronectin (Millipore) at 37°C, 5% CO_2_ in N2B27 medium (Gibco) supplemented with GlutaMAX‐I (2 mmol/L; Gibco), beta‐mercaptoethanol (beta‐ME, 0.01 mmol/L; Gibco), leucocyte inhibitory factor (LIF, 20 ng/mL; R&D systems), CHIR99021 (3 μmol/L; R&D systems) and PD0325901 (1 μmol/L; R&D systems). The outgrowths of the embryos formed bowl‐like ESC colonies in less than a week. The ESC colonies were then picked and propagated by passaging every 2 days.

### 2.8 Cell proliferation assay

The ESCs at the logarithmic growth phase were trypsinized using 0.25% Trypsin‐EDTA (Gibco) and plated on 48‐well plates at a density of 5000 cells/well and cultured for 1‐5 days. Cell proliferation was examined using the Cell Counting Kit‐8 (CCK‐8) kit (Solarbio), following the manufacturer's instructions. Briefly, 1/10 volume of CCK‐8 reagent was added into the culture medium in each well and incubated for 2 hours. The absorbance of each well at 450 nm was measured using a BioTek spectrophotometer.

### 2.9 Western blotting

Western blotting was performed as described previously.^25^ Briefly, ESCs were lysed with RIPA lysis buffer (0.5% NP‐40, 50 mmol/L Tris‐HCl, 150 mmol/L NaCl, 1 mmol/L EDTA, pH = 8) supplemented with proteinase and phosphatase inhibitors (Thermo Fisher). The cell lysates were subjected to sodium dodecyl sulphate gel electrophoresis (SDS‐PAGE) at 110 V for 2 hours. The proteins on the gel were transferred to a nitrocellulose membrane (Thermo Fisher). The membrane was then blocked with 5% bovine serum albumin (BSA) prepared in PBS. The membrane was probed with appropriate primary antibodies and corresponding secondary antibodies. The proteins were detected in a LI‐COR (ODYSSEY CLx) exposing equipment. Antibodies used in this assay are listed in Table S2.

### 2.10 Apoptosis assay

The apoptosis rate of ESCs was examined using the FITC Annexin V Apoptosis Detection Kit II (BD Biosciences), following the manufacturer's instructions. Briefly, logarithmic phase ESCs were trypsinized with 0.25% Trypsin‐EDTA (Gibco), washed twice with cold phosphate buffer saline (PBS, pH = 7.4). The cells were resuspended in 1× Binding buffer at a concentration of 1 × 10^6^ cells/mL. Next, 100 µL of the cell suspension (1 × 10^5^ cells) was transferred to a 5‐mL culture tube and incubated with 5 µL of FITC Annexin V and 5 µL of propidium iodide (PI) for 15 minutes at room temperature (25°C) in the dark. The cells were subjected to fluorescence‐activated cell sorting (FACS) using a BD (MoFlo XDP) flow cytometer. FACS data were analysed using Flow Jo software (v10.0.7).

### 2.11 Cell cycle analysis

The cell cycle of the ESCs was analysed using the BD Pharmingen 488 EdU Click Proliferation Kit, following the manufacturer's instructions. EdU was incubated with the logarithmic phase ESCs cultured in 6‐well plates for 30 minutes. 1 × 10^7^ cells were harvested and resuspended in 1 mL BD Pharmingen Stain Buffer. The cells were then subjected to centrifugation at 800 × *g* for 2 minutes. After centrifugation, the supernatant was removed and the cell pellet was resuspended with 100 μL fixative solution and incubated for 15 minutes at room temperature of fixation. The cells were washed once with 1 mL BD Pharmingen Stain Buffer, resuspended in 100 μL of saponin‐based permeabilization buffer and incubated with 500 μL of EdU detection cocktail for 30 minutes at room temperature in the dark. The treated cells were washed twice with 2 mL of 1× saponin‐based permeabilization buffer and resuspended in 500 μL of 1× PBS containing 1 μg/mL of Hoechst33342 (Invitrogen) before FACS analysis.

### 2.12 Single cell gel electrophoresis assay

Single cell gel electrophoresis assay was performed using the Trevigen's Comet Assay Kit, following the manufacturer's instructions. The logarithmic phase ESCs were harvested and resuspended in PBS at a concentration of 1 × 10^5^ cells/mL and were embedded in 37°C molten LMAgarose at a ratio of 1:10 (v/v). The LMAgarose‐cell mixture was plated onto the comet slides evenly at 50 μL/well. The slides were placed flat at 4°C in the dark for 10 minutes to allow the solidification of the gel. Next, the slides were sequentially immersed in 4°C lysis solution for 30‐60 minutes and in freshly prepared 4°C alkaline unwinding solution for 1 hour in the dark. The slides were then subjected to electrophoresis in alkaline electrophoresis solution at 25 V for 40 minutes at 4°C. After electrophoresis, the slides were washed twice with double distilled water (ddH_2_O), once with 75% ethanol and dried at 37°C for 10‐15 minutes. The dried slides were then stained with 100 μL of diluted SYBR Gold (1:10 000 in TE buffer, Invitrogen) and photographed using a Carl Zeiss LSM 780 confocal system. The images were analysed on a CaspLab comet assay software (version 1.2.3b1).

### 2.13 IP‐MS and Co‐IP

Immunoprecipitation (IP) was performed using the Pierce Crosslink Magnetic IP/Co‐IP Kit (Thermo Fisher), following the manufacturer's instructions with minor modifications. Briefly, 10 μg of anti‐GFP antibody (Abcam, ab290) or IgG antibody was bound to 50 μL of Pierce Protein A/G Magnetic Beads. The coupled antibodies were cross‐linked to the magnetic beads using the DSS cross‐linker. The ESCs (1 × 10^7^) were lysed with 500 μL of ice‐cold IP lysis/wash buffer and incubated with the antibody‐bead mixture at 4°C overnight. The beads were collected using a magnetic stand and washed with IP lysis/wash buffer for five times followed by elution with 100 µL of elution buffer. The eluted proteins were subjected to mass spectrometry or western blot analysis.

### 2.14 RNA‐Seq and bioinformatic analysis

RNA isolation and RNA‐Seq were performed as described previously.^25^ Briefly, 5 × 10^6^ ESCs were harvested and lysed using 1 mL TRIzol reagent (Invitrogen). The RNA was purified using 1:5 (v/v) chloroform (Sinopharm Chemical Reagent Beijing Co., Ltd) and precipitated by centrifugation using 1:1 (v/v) isopropanol alcohol (Sinopharm Chemical Reagent Beijing Co., Ltd). The RNA pellet was dissolved in RNase‐free ddH_2_O (Invitrogen) at a concentration of about 1 μg/μL. The cDNA libraries were constructed using the TruSeq RNA Sample Prep Kit with 10 μg of the isolated RNA per sample. All samples were sequenced by an Illumina HiSeq‐X TEN sequencing system with paired‐end 150‐bp read length. For the data analysis, the mm10 version annotation for mouse genome were used. Clean RNA‐Seq reads were mapped by HISAT2 with default settings (version 2.1.0). Reads with unique genome location were used for splicing analysis. rMATS were used to identify differentially splicing events between wild‐type and *Rbm14* knockout samples. The thresholds were set at D‐value of percent spliced in (ΔPSI) = 10% and false discovery rate (FDR) = 0.05. The sashimi plots were produced by rmats2sashimiplot (https://github.com/Xinglab/rmats2sashimiplot/). The gene ontology analysis was performed by DAVID (version 6.8), and the figures were plotted by ggplot2.

### 2.15 PAR‐CLIP‐Seq and bioinformatic analysis

PAR‐CLIP experiments were conducted as described.^26^ Briefly, *Gfp‐Rbm14* mESCs were pulsed with 100 μmol/L 4‐thiourdine (4SU, Sigma) overnight. After irradiation with UV at 365 nm, RBM14/RNA complexes were immunoprecipitated with the anti‐GFP antibody and protein A magnetic beads (Thermo). After RNase T1 treatment, the immunoprecipitates were resolved on 4%‐12% LDS gel with Mops buffer (Life Science Tech). RNA on the gel was visualized with SYBR green I staining on the PhosphorImager (GE). RNA bound to the RBM14 protein was extracted from the gel, freed from protein by digestion with proteinase K, purified, converted into cDNA and sequenced by Illumina X Ten. The PAR‐CLIP‐Seq data were analysed as described in previous paper.^27^ In brief, The PAR‐CLIP‐Seq reads of the RBM14 protein were trimmed with the Cutadapt software (version 2.4).^28^ Reads that were less than 18 nt in length or contained an ambiguous nucleotide were discarded. The remained reads were aligned to the mouse genome (mm10), with parameters recommended by PARalyzer (v1.5)^29^ by the Bowtie algorithm (version 1.2.3).^30^ Clusters were obtained by PARalyzer (v1.5) with the default parameters.

### 2.16 Quantitative PCR and semi‐quantitative PCR

RNA was reverse transcribed into cDNA using the Reverse Transcription System (Promega), following the manufacturer's instructions. Briefly, 1 μg RNA were mixed with 1 μL random primers and 1 μL oligo primers and then added with RNase‐free ddH_2_O up to 13.5 μL, then the RNA was annealed by incubation at 70°C for 5 minutes followed by incubation on ice for 2 minutes. After then 1 μL MLV reverse transcriptase, 4 μL 5× reaction buffer, 0.5 μL RNasin and 1 μL dNTP were added in the annealed RNA. RNA was reverse transcribed in 42°C for 1 hour followed by heat inactivation at 85°C for 5 minutes. For quantitative PCR, 0.2 μL cDNA were used as templates for PCR using SYBR Green PCR Master Mix (Toyobo) on an Agilent real‐time fluorescence quantitative PCR instrument (Mx3000P). The formula −2^ΔΔ^ *^C^* ^T^ was used to calculate the relative expression of *Rbm14*. For semi‐quantitative PCR, 1 μL cDNA was then used as templates for PCR using the Vazyme 2 × PCR Master Mix. The PCR products were then analysed using the agarose gel electrophoresis system (Bio‐Rad). Primers for quantitative PCR and semi‐quantitative PCR were listed in Table S1.

### 2.17 Statistical analysis

All statistical analyses (unless stated otherwise) were performed using the R package for statistical computing. For experimental data quantification, Students’ *t*‐test was applied using the GraphPad Prism 6 software. The error bar represents the standard error of mean (SEM) (unless stated otherwise). The difference was considered statistically significant when the *P* value was <.05.

## 3 RESULTS

### 3.1 *Rbm14* knockout leads to embryonic lethality in mice

To investigate the role of *Rbm14* in mouse embryonic development, we generated a *Rbm14* knockout allele through CRISPR/Cas9‐mediated non‐homologous end joining (NHEJ) (Figure S1A). The Cas9 mRNA and a single guide RNA (sgRNA) targeting the first exon of *Rbm14* just downstream of the start codon were introduced into the zygote‐stage mouse embryos through microinjection. Finally, a male founder mouse harbouring a *Rbm14* knockout allele was obtained. The *Rbm14* knockout allele contains a 68 bp deletion in the target site and results in frame shift and premature termination of translation at the N‐terminus of the RBM14 protein. The founder mouse was crossed with wild‐type mice (*Rbm14* ^+/+^) to generate *Rbm14* ^+/−^ heterozygous pups, the genotypes of the pups were detected by polymerase chain reaction (PCR, Figure S1B) and confirmed by Sanger sequencing (Figure S1C). The *Rbm14* ^+/−^ mice were fertile and showed no abnormalities when compared to their *Rbm14* ^+/+^ counterparts. Next, we investigated the role of *Rbm14* in mouse embryonic development using various mating strategies: ♀ *Rbm14* ^+/+^ × ♂ *Rbm14* ^+/−^; ♀ *Rbm14* ^+/−^ × ♂ *Rbm14* ^+/+^ and ♀ *Rbm14* ^+/−^ × ♂ *Rbm14* ^+/−^ (Table 1). The proportion of pups with different genotypes obtained was consistent with the Mendelian ratio by crossing *Rbm14* ^+/+^ female mice with the *Rbm14* ^+/−^ male mice (46.1% for *Rbm14* ^+/+^ to 53.9% for *Rbm14* ^+/−^) or by crossing *Rbm14* ^+/−^ female mice with the *Rbm14* ^+/+^ male mice (51.5% for *Rbm14* ^+/+^ to 48.5% for *Rbm14* ^+/−^). However, no *Rbm14* ^−/−^ mouse was born when we interbred the *Rbm14* ^+/−^ male and female mice. The ratio of *Rbm14* ^+/+^ to *Rbm14* ^+/−^ pups obtained in this mating was about 1:2 (32.9%‐67.1%), which indicated embryonic lethality of the homozygous *Rbm14* knockout mice.

**Table 1:** *Rbm14* knockout in mice is embryonic lethal

Genotype	Number of progenies		
	♀ *Rbm14* ^+/+^ × ♂ *Rbm14* ^+/−^	♀ *Rbm14* ^+/−^ × ♂ *Rbm14* ^+/+^	♀ *Rbm14* ^+/−^ × ♂ *Rbm14* ^+/−^
*Rbm14* ^+/+^	65 (46.1%)	69 (51.5%)	51 (32.9%)
*Rbm14^±^*	76 (53.9%)	65 (48.5%)	104 (67.1%)
*Rbm14* ^−/−^	0	0	0
Mendelian ratio	1:1:0	1:1:0	1:2:1

Note

Quantification of pups with different genotypes by three different mating strategies: ♀ *Rbm14* ^+/+^ × ♂ *Rbm14* ^+/−^; ♀ *Rbm14* ^+/−^ × ♂ *Rbm14* ^+/+^ and ♀ *Rbm14* ^+/−^ × ♂ *Rbm14* ^+/−^. No *Rbm*14^−/−^ pups were produced by interbreeding of *Rbm14* ^+/−^ male and female mice, indicating that homozygous deletion of *Rbm14* ^+/−^ results in embryonic lethality. More than 10 litters of pups were genotyped and quantified in each group (for ♀ *Rbm14* ^+/+^ × ♂ *Rbm14* ^+/−^, n = 141; for ♀ *Rbm14* ^+/−^ × ♂ *Rbm14* ^+/+^, n = 134 and for ♀ *Rbm14* ^+/−^ × ♂ *Rbm14* ^+/−^, n = 155).

### 3.2 *Rbm14* knockout inhibits gastrulation during early embryonic development in mice

Next, we examined the role of *Rbm14* in pre‐implantation embryonic development. We first detected the expression pattern of *Rbm14* during early pre‐implantation embryonic development. Results showed that *Rbm14* mRNA was abundantly deposited in mouse zygotes and 2‐cell blastomeres. While the expression of *Rbm14* was downregulated during embryogenesis from the 4‐cell stage to the blastocyst stage (Figure S2A). We then collected the blastula‐stage embryos after interbreeding *Rbm14* ^+/−^ male and female mice at E4.0 stage by flushing the uteruses of the pregnant female mice. In the late pre‐implantation stage, the ICM of the embryo divides into NANOG^+^ and OCT4^+^ epiblast and the OCT4^+^ primitive endoderm. Immunostaining for NANOG and OCT4 revealed no substantial defects in the pre‐implantation blastocyst stage embryos with the knockout of *Rbm14* (Figure S2B). Genotype analysis of the embryos revealed that the proportion of embryos with different genotypes was in accordance with the Mendelian ratio (20.7% for *Rbm14* ^+/+^ to 55.2% for *Rbm14* ^+/−^ to 24.1% for *Rbm14* ^−/−^, Figure S2C). Our findings indicated that although *Rbm14* was highly expressed during the pre‐implantation stage, *Rbm14* knockout could support the development of mouse embryos, at least, from zygotes to blastocysts.

**Figure 1:**
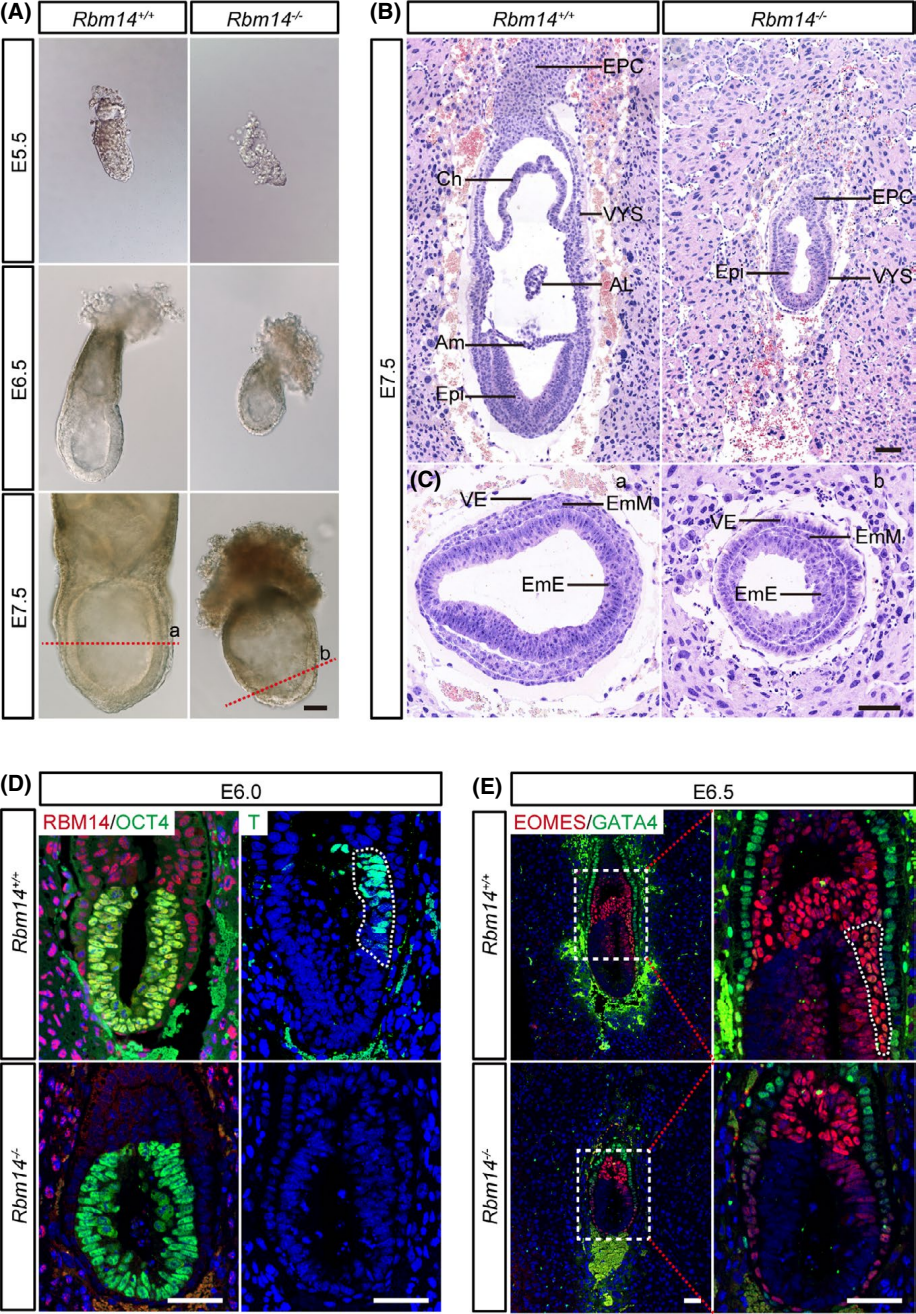
*Rbm14* knockout inhibits gastrulation during early mouse embryonic development. A, Representative images showing the micromorphological structures of both wild type and *Rbm14* knockout embryos obtained after interbreeding of *Rbm14* ^+/−^ male and female mice at E5.5, E6.5 and E7.5 stages. Scale bar, 100 μm. The embryos were dissected from the uterus of pregnant female mice and imaged and then subjected to genotyping. The *Rbm14* knockout embryos are smaller in size when compared to their wild type counterparts. B, C, Haematoxylin‐eosin (H&E) staining of wild type and *Rbm14* knockout embryos at E7.5 stage shows histological abnormalities in the *Rbm14* knockout embryos. B, Longitudinal sections of H&E staining show that structures such as amnion, chorion and allantois are missing in the knockout embryo (B). H&E staining of different germ layers demonstrates that the knockout embryo exhibits marked cell number loss, especially in the embryonic region (C). Panels a and b are corresponding to the cut site indicated by the red dotted lines in (A). Scale bar, 100 μm. AL, allantois; Am, amnion; Ch, chorion; EmE, embryonic ectoderm; EmM, embryonic mesoderm; EPC, ectoplacental cone; Epi, epiblast; VE, visceral endoderm; VYS, visceral yolk sac. D, Representative immunofluorescent images of both wild type and *Rbm14* knockout embryos at E6.0 stage for RBM14 (red), the epiblast marker OCT4 (green) and the mesoderm maker T (green). The nuclei were counterstained with 4′,6‐diamidino‐2‐phenylindole (DAPI) and are shown in blue. Scale bar, 50 μm. The posterior region expressing the mesoderm‐related *T* gene is outlined by white dotted lines in the wild‐type embryo. The absence of RBM14 staining in the knockout embryo confirms complete depletion of RBM14 protein. Expression of the mesoderm‐related gene *T* is suppressed in the knockout embryo upon knockout of *Rbm14*. E, Representative immunofluorescent images of both wild type and *Rbm14* knockout embryos at E6.5 for the primitive streak marker EOMES (red) and the visceral endoderm marker GATA4 (green). The nuclei were counterstained with DAPI and are shown in blue. Scale bar, 50 μm. The primitive streak of the wild‐type embryo is outlined by white dotted lines. The absence of the primitive streak in the knockout embryo indicates the disruption of embryonic development at the early gastrulation stage upon knockout of *Rbm14*. For either wild type or knockout embryos at each stage in these histological analyses, n = 3

We then dissected embryos at E5.5, E6.5 and E7.5 by mating *Rbm14* ^+/−^ male and female mice and observed abnormalities in size and morphology in the *Rbm14* knockout embryos as early as E5.5 (Figure 1A and Figure S3A,B). Haematoxylin‐eosin (H&E) staining at E7.5 exhibited the absence of late gastrulation stage structures such as chorion, amnion and allantois in the *Rbm14* knockout embryos (Figure 1B). The knockout embryos also showed lower cell density in the epiblast and embryonic mesoderm. While in the visceral endoderm of the knockout embryos, cells were piled up as a consequence of the cell number loss in the embryonic region (Figure 1C). We then analysed the developmental defects in *Rbm14* ^−/−^ embryos by immunostaining. At the early post‐implantation stage of E6.0, we detected suppressed expression of the mesoderm‐related gene *T* upon knockout of *Rbm14*. The absence of RBM14 staining signal in the *Rbm14* ^−/−^ knockout embryos confirmed complete depletion of the RBM14 protein (Figure 1D). The suppressed expression of *T* in the *Rbm14* ^−/−^ embryos lasted till the E7.5 stage (Figure S3C). During mouse embryogenesis, the primitive streak (PS) forms at the posterior face of the developing embryo at about E6.5. The formation of the PS marks the initiation of gastrulation. The absence of PS in the *Rbm14* ^−/−^ embryos was detected by EOMES staining at E6.5 (Figure 1E). Consistent with the H&E staining results, GATA4 staining revealed aberrant accumulation of visceral endoderm cells outside the embryonic region of the *Rbm14* ^−/−^ embryos (Figure S3D). Our findings demonstrated that *Rbm14* knockout resulted in developmental arrest during gastrulation, indicating a critical role of *Rbm14* in early development of mouse embryos.

### 3.3 *Rbm14* knockout induces apoptosis and cell cycle arrest in the post‐implantation epiblast

**Figure 2:**
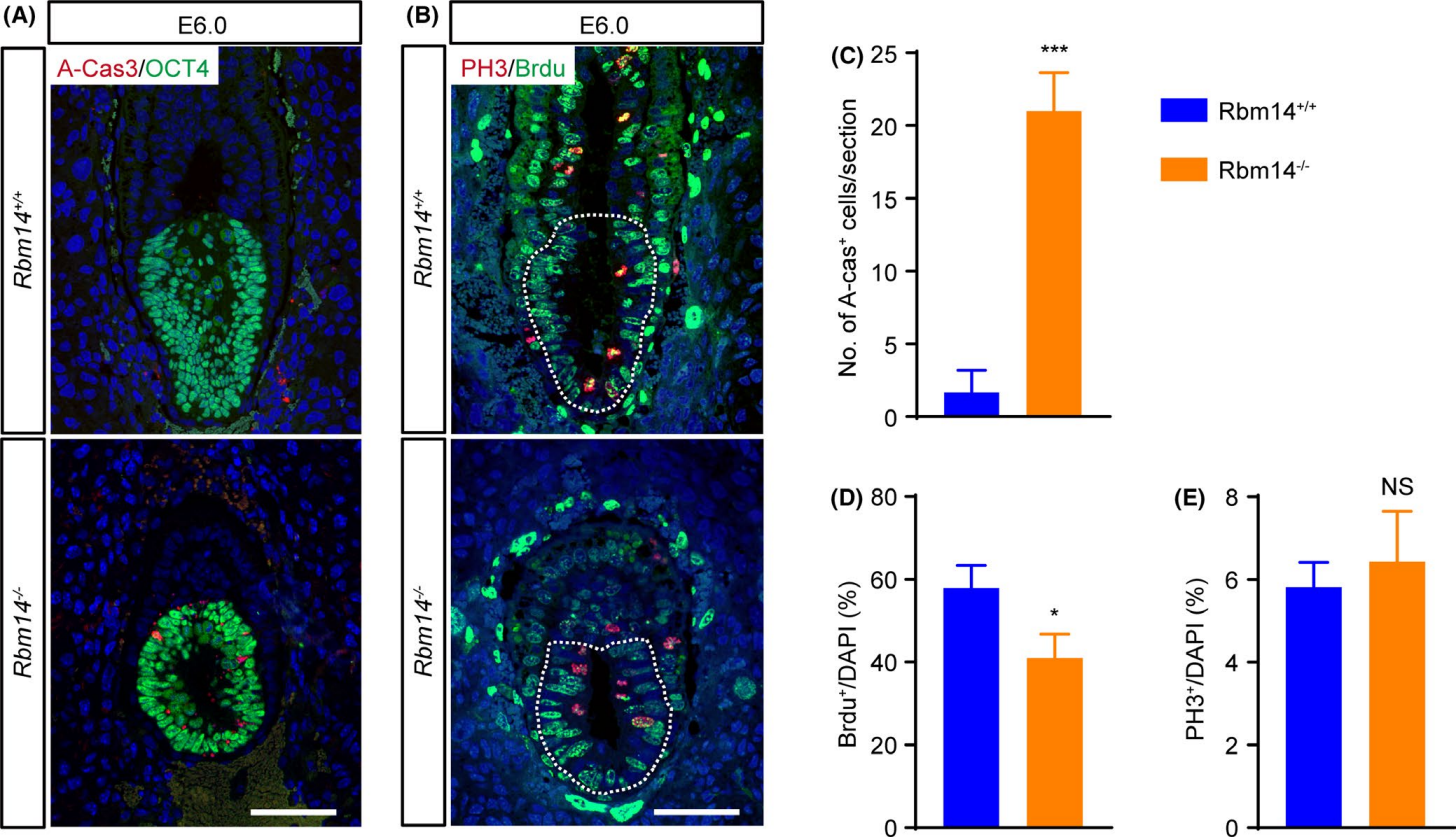
*Rbm14* knockout induces apoptosis and cell cycle arrest of post‐implantation epiblast. A, Representative immunofluorescent images of both wild type and *Rbm14* knockout embryos at E6.0 stage for the apoptosis marker active caspase‐3 (red) and the epiblast marker OCT4 (green). The nuclei were counterstained with 4′,6‐diamidino‐2‐phenylindole (DAPI) and are shown in in blue. Scale bar, 50 μm. Elevated apoptosis is detected in the *Rbm14* knockout embryo, and most of the active caspase‐3^+^ cells co‐express OCT4. B, Representative immunofluorescent images of both wild type and *Rbm14* knockout embryos at E6.0 stage for the G2/M phase marker histone H3 (phospho S10) (red) and BrdU (green). The nuclei were counterstained with DAPI and are shown in blue. The epiblast regions of the embryos are outlined with white dotted lines. Scale bar, 50 μm. BrdU were injected into the pregnant female mice intraperitoneally and were incorporated into the mitotic cells at S phase. The embryos were dissected from the uterus of pregnant female mice 1 h later. C, Quantification of the immunostaining shown in (A). Three sections for each group were analysed. Data are shown as mean ± SEM. ***P < .001, Student's *t*‐test. D, E, Quantification of the immunostaining shown in (B). The proportion of BrdU^+^ cells decreased in the *Rbm14* knockout E6.0 epiblast. Three sections from three different embryos for each group were analysed (n = 3). Data are shown as mean ± SEM. *P < .05, Student's *t*‐test

The reduced size and loss of cell density in the *Rbm14* knockout embryos indicated elevated cell apoptosis activities. Thus, we performed immunostaining for the cell apoptosis marker, active caspase‐3. We detected more active caspase‐3^+^ cell in the *Rbm14* ^−/−^ embryos at the E6.0 stage. Additionally, co‐staining of the active caspase‐3 with OCT4 demonstrated that cell apoptosis mainly occurs in the epiblast cells (Figure 2A,C). Bromodeoxyuridine (BrdU) staining also detected cell cycle arrest in the *Rbm14* ^−/−^ epiblast cells (Figure 2B,D,E). These results demonstrated that the developmental defects of the *Rbm14* ^−/−^ embryos were due to cell apoptosis and cell cycle arrest of the post‐implantation epiblast.

### 3.4 *Rbm14* knockout causes apoptosis and cell cycle arrest in mouse embryonic stem cells

**Figure 3:**
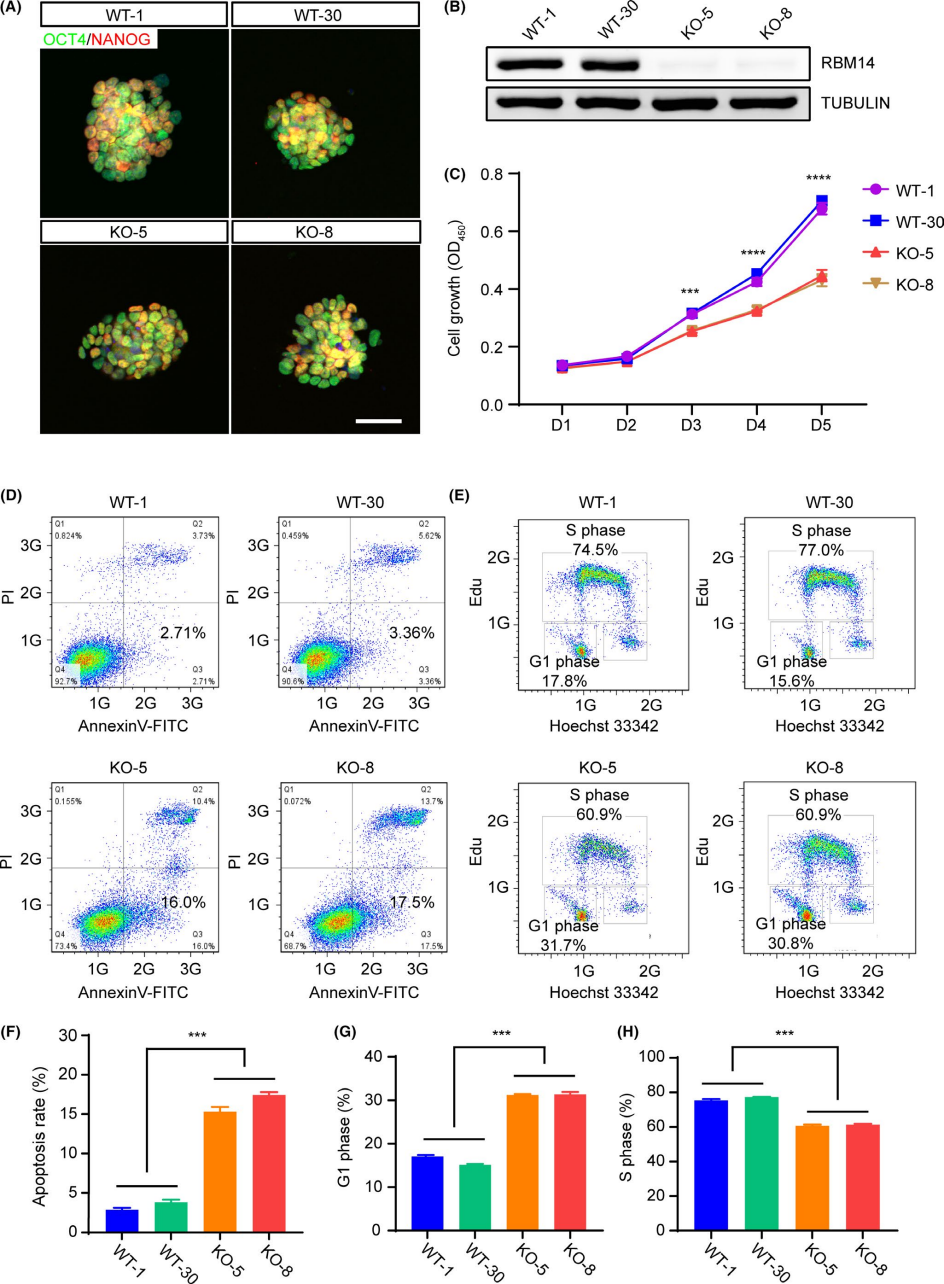
*Rbm14* knockout causes apoptosis and cell cycle arrest in mouse embryonic stem cells (ESCs). A, Representative immunofluorescent images of both wild type and *Rbm14* knockout mouse ESCs for pluripotent markers NANOG (red) and OCT4 (green). Mouse ESC cell lines were isolated from the ICM of E3.5 embryos. The nuclei were counterstained with 4′,6‐diamidino‐2‐phenylindole (DAPI) and are shown in blue. Scale bar, 20 μm. Three independent biological repeats were conducted. B, Western blot confirms the depletion of RBM14 in *Rbm14* knockout ESCs. Three independent biological repeats were conducted and one representative image is shown. C, Cell counting kit‐8 assay revealed decreased proliferation rate of *Rbm14* knockout ESCs. The OD_450_ values from three independent experiments were analysed (n = 3). Data are shown as mean ± SEM. ***P < .001, ****P < .0001, Student's *t*‐test. D, Fluorescence‐activated cell sorting (FACS) analysis after Annexin V‐PI staining reveals elevated apoptosis rate in the *Rbm14* knockout ESCs. Annexin V^+^/PI^−^ cells represent early apoptosis. E, FACS analysis after 5‐ethynyl‐2′‐deoxyuridine (EdU)‐Hoechst33342 staining reveals cell cycle arrest in the *Rbm14* knockout ESCs. The cells were incubated with EdU for 1 h before staining. The cells incorporated with EdU represent the S phase of the cell cycle. F, Quantification of the FACS analysis in (D). The ratio of Annexin V^+^/PI^−^ cells from three independent experiments were analysed. Data are shown as mean ± SEM (n = 3). ***P < .001, Student's *t*‐test. G, H, Quantification of the FACS analysis in (E). The ratio of G1 phase and S phase cells from three independent experiments was analysed. Data are shown as mean ± SEM (n = 3). ***P < .001, Student's *t*‐test

To further investigate the underlying mechanisms for the requirement of *Rbm14* in early mouse embryonic development, we derived ESCs from the E3.5 *Rbm14* ^+/+^ and *Rbm14* ^−/−^ blastula stage embryos. The expression of pluripotency‐related genes, such as *Nanog* and *Oct4* were evaluated in these embryos by immunostaining. Our results revealed that both *Rbm14* ^+/+^ and *Rbm14* ^−/−^ ESCs expressed these pluripotent genes in the protein level (Figure 3A). We also confirmed the depletion of RBM14 protein in the *Rbm14* knockout ESCs by western blotting (Figure 3B). Although no obvious developmental defect was observed in the pre‐implantation *Rbm14* ^−/−^ embryo as described above, we detected decreased proliferation rate in the *Rbm14* ^−/−^ ESCs (Figure 3C). We then analysed the apoptosis of *Rbm14* ^−/−^ ESCs. We detected markedly elevated apoptosis in the *Rbm14* ^−/−^ ESCs (Figure 3D,F). Moreover, cell cycle analysis using 5‐ethynyl‐2′‐deoxyuridine (EdU) labelling revealed that the G1 phase was delayed in the *Rbm14* ^−/−^ ESCs (Figure 3E,G,H). These results demonstrated that the knockout of *Rbm14* results in apoptosis and cell cycle arrest in the mouse ESCs, which was consistent with the proliferation defects observed in the epiblast of the *Rbm14* ^−/−^ early mouse embryo.

### 3.5 *Rbm14* is required for maintenance of genomic DNA integrity

**Figure 4:**
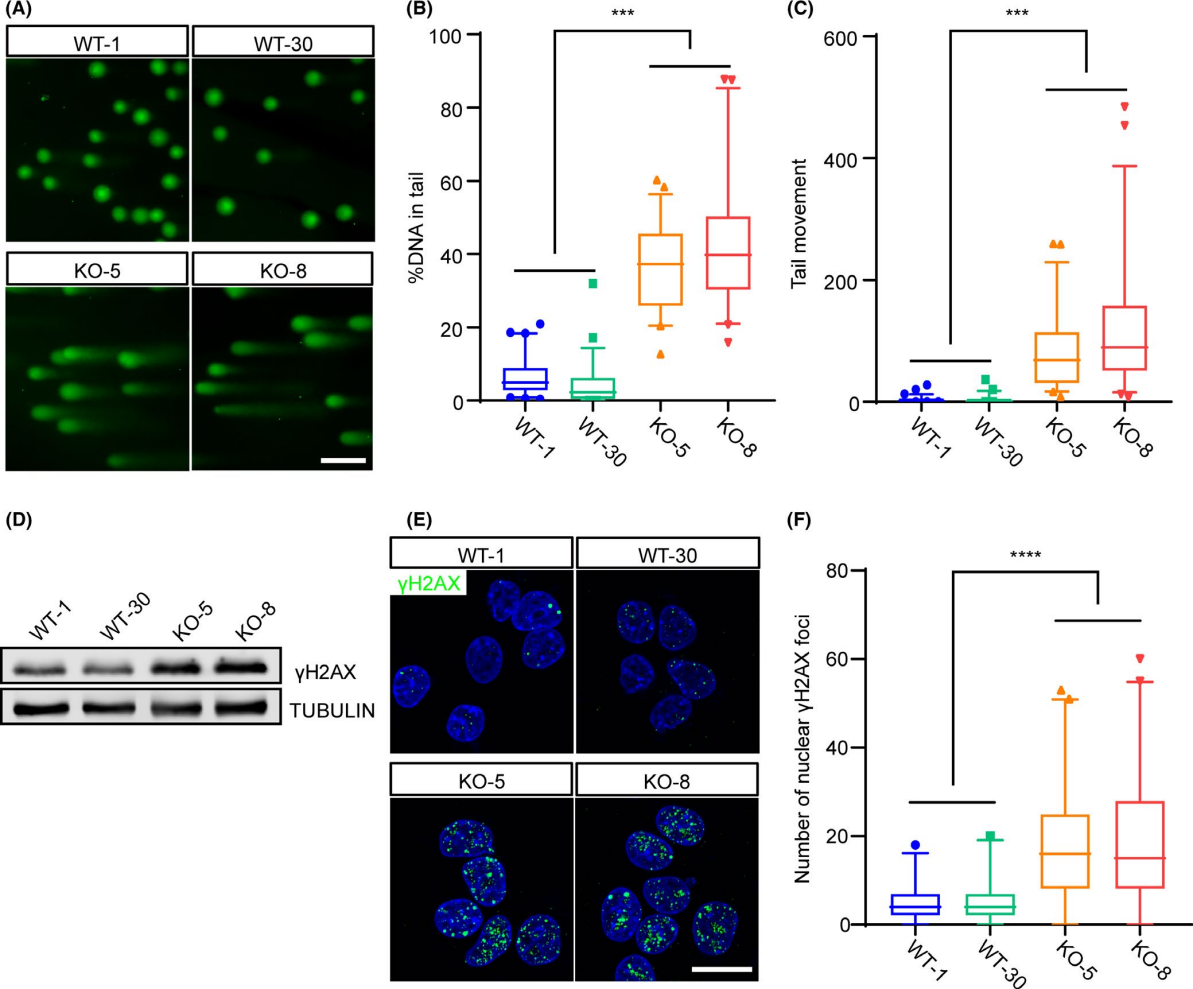
*Rbm14* knockout causes DNA damage in mouse embryonic stem cells. A‐C, Comet assay reveals severe DNA damage in the *Rbm14* knockout embryonic stem cells (ESCs). Genomic DNA was stained with SYBR Gold and imaged after single cell electrophoresis (A). Scale bar, 50 μm. Quantification for the percent of DNA in the comet tail (B). Quantification for the length of the tail movement (C). Data are shown as mean ± SEM (for WT‐1, n = 69; for WT‐30, n = 51; for KO‐5, n = 51 and for KO‐8, n = 45.) ***P < .001, Student's *t*‐test. D, Western blot shows elevated γH2AX protein level in the *Rbm14* knockout ESCs. E, Representative immunofluorescent images of wild type and knockout ESCs for the DNA damage marker γH2AX (green). The nuclei were counterstained with 4′,6‐diamidino‐2‐phenylindole (DAPI) and are shown in blue. Increased γH2AX foci are detected in *Rbm14* knockout ESCs. Scale bar, 10 μm. F, Quantification of the γH2AX foci in the immunofluorescent staining in (D). More than 50 cells were analysed in each group. Data are shown as mean ± SEM (for WT‐1, n = 189; for WT‐30, n = 190; for KO‐5, n = 211 and for KO‐8, n = 208.) **P < .01, Student's *t*‐test

For proliferating cells, DNA damage is an intense stress that may cause cell death and cell cycle arrest.^31^, ^32^ To investigate whether *Rbm14* participates in the maintenance of DNA integrity during early mouse embryogenesis, we performed single cell gel electrophoresis analysis (Figure 4A) and revealed that the *Rbm14* ^−/−^ ESCs exhibited more fragmented DNA (Figure 4B) and longer comet tails (Figure 4C) than their wild‐type counterparts. This result demonstrated severe DNA damage in the *Rbm14* knockout ESCs. Additionally, western blotting revealed that the expression of γH2AX, a DNA damage marker, was elevated in the *Rbm14* ^−/−^ ESCs (Figure 4D). Immunostaining also revealed increased number of γH2AX foci in the nuclei of *Rbm14* ^−/−^ ESCs (Figure 4E,F). The accumulation of DNA damage in the *Rbm14* knockout ESCs indicated that RBM14 was required for maintaining the genome integrity during early embryonic development.

### 3.6 *Rbm14* regulates alternative splicing of DDR‐related genes

**Figure 5:**
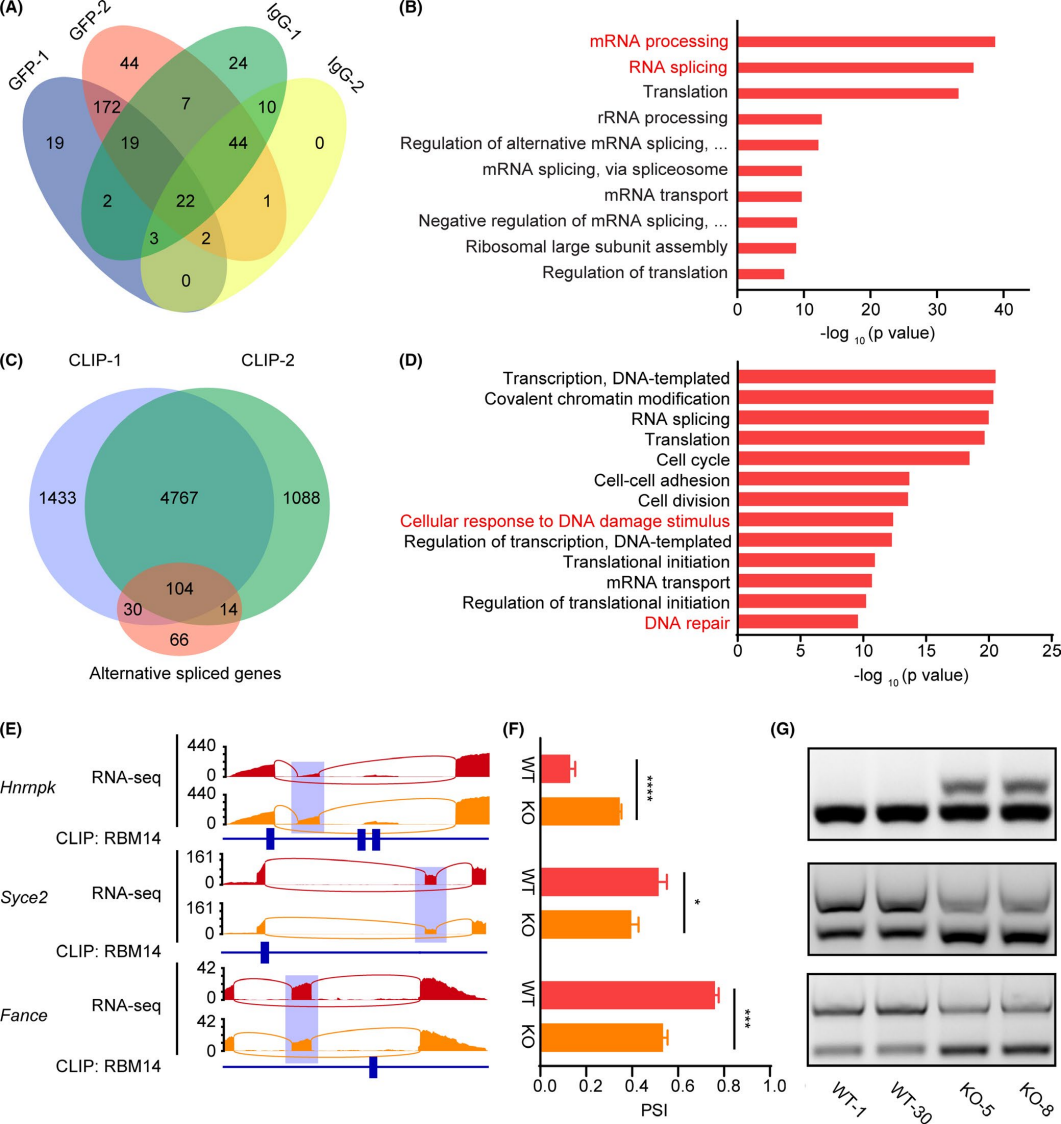
*Rbm14* regulates alternative splicing of DDR‐related genes. A, Venn diagram showing the number of proteins in each group of immunoprecipitation mass spectrometry (IP‐MS). Two independent replicates were used and identified 172 proteins interacting with RBM14. B, GO analysis for proteins interacting with RBM14 shows enriched RNA processing associated terms. C, Venn diagram showing the identified numbers of genes with alternatively spliced exons and with RBM14 binding peaks in the PAR‐CLIP‐Seq assay. Two independent replicates were conducted. Of all the 214 genes with alternatively spliced exons, 48.6% have RBM14 binding peaks on their mRNA. D, GO analysis for genes having RBM14 binding peaks on their mRNA. E, Representative sashimi plots displaying RNA‐Seq reads coverage and the RBM14 binding sites (indicated by blue rectangular blocks) of the alternatively spliced genes associated with DDR. Alternatively, spliced regions are indicated by semitransparent blocks. F, Calculated PSI value of the alternatively spliced genes associated with DDR in both wild type and *Rbm14* knockout embryonic stem cells (ESCs). Data are shown as mean ± SEM. Three cell lines for each genotype were sequenced and analysed (n = 3). *P < .05, **P < .01, ***P < .001, ****P < .0001, Student's *t*‐test. G, Semi quantitative‐PCR confirms the altered splicing of DDR associated genes. PSI, percent spliced in

Previous studies have reported that *Rbm14* may participate in NHEJ DNA repair by interacting with Ku80 in glioblastoma (GBM) and HeLa cells.^21^, ^22^ To further investigate the role of *Rbm14* in DDR in ESCs and in early embryonic development, we performed Immunoprecipitation‐Mass Spectra (IP‐MS) with a GFP tagged *Rbm14* (*Gfp*‐*Rbm14*) ESC cell line (Figure S4A) and identified 172 proteins that interacted with RBM14 protein (Figure 5A). Gene ontology (GO) term analysis revealed that most of the proteins interacting with RBM14 were enriched in RNA processing related processes, particularly in “RNA splicing” (Figure 5B), indicating that *Rbm14* was involved in regulating mRNA alternative splicing. We then confirmed the interaction of RBM14 protein with splicing associated proteins such as SF3B1 and SF3B3 by co‐immunoprecipitation (Co‐IP). Interestingly, we detected no interaction of RBM14 with Ku80 (Figure S4B), indicating that RBM14 participated in DDR of ESCs in quite a different way from that in HeLa cells. We then performed RNA sequencing for *Rbm14* ^+/+^ and *Rbm14* ^−/−^ ESCs and analysed the altered splicing events. We detected 330 alternative splicing events under the threshold of 10% percent spliced in (PSI) changes (Figure S4C). GO analysis showed that many of the alternatively spliced genes were enriched in the term “Cellular response to DNA damage stimulus” (Figure S4D). To identify the direct interaction of RBM14 with the transcripts of alternatively spliced genes, we performed PAR‐CLIP‐Seq and obtained 4871 genes whose transcripts were bound by RBM14. Two independent replicates were conducted with a large overlap between the RBM14‐binding transcripts. Of all the 214 genes with alternatively spliced exons, 48.6% have RBM14 binding peaks on their mRNA (Figure 5C). GO analysis showed that genes having RBM14 binding peaks on their mRNA were enriched in terms such as “Cellular response to DNA damage stimulus” and “DNA repair”, indicating that RBM14 might directly bind to the transcripts of DDR‐associated genes and regulate their splicing process (Figure 5D). We then confirmed the altered splicing of genes related to DDR, such as *Hnrnpk*,* Syce2* and *Fance* in *Rbm14* knockout ESCs by semi‐quantitative PCR. We also found that all these genes harboured RBM14 binding peaks on the adjacent exons or introns of the alternatively spliced exons (Figure 5E‐G). These results demonstrated that *Rbm14* was involved in genomic DNA maintenance by regulating the alternative splicing of DDR‐related genes.

## 4 DISCUSSION

The early stage development of mouse embryo initiates with the first cleavage of the fertilized egg and proceeds till gastrulation at about E7.5. At the end of the gastrulation stage, three distinct lineages are established: mesoderm, endoderm and ectoderm. During the early phase of embryonic development, the number of cells increases drastically from 1 to more than 10 thousand by proliferation. Additionally, the number of cell types in the embryo increases by fine‐tuned cell differentiation. Several studies in the last few decades have identified key molecular players and mechanisms orchestrating these activities during early mouse embryogenesis. In this study, we demonstrated that RBM14, an RRM family protein, regulates early mouse embryogenesis through alternative splicing. Homozygous knockout of *Rbm14* in mice was embryonic lethal. We also demonstrated that *Rbm14* knockout results in apoptosis and cell cycle arrest of the post‐implantation epiblast cells, which subsequently leads to disruption of gastrulation.

Alternative splicing is an essential mechanism to increase the complexity and diversity of the mammalian transcriptome. Alternative splicing of pre‐mRNA enables efficient gene expression as one single gene may contain coding information for many proteins that differ in their structures and functions.^33^ A genome‐wide analysis of the alternative isoform expression revealed that alternative splicing and isoform‐specific expression were frequently observed during the development of early mouse embryo.^34^ Previous studies have reported the implication of alternative splicing mediated by RRM proteins in early embryonic development.^35^, ^36^, ^37^ RBM14 was originally reported to be a general nuclear coactivator that interacts with thyroid hormone receptor‐binding protein (TRBP) to regulate the activation of gene transcription.^38^ Further studies revealed roles of *Rbm14* in preserving the mitotic spindle integrity^20^ and regulation of DNA virus‐mediated innate immune response.^39^ However, few studies have evaluated the role of *Rbm14* in alternative splicing regulation.^19^ In this study, we recognized the interaction of RBM14 with core splicing factors such as SF3B1 and SF3B3 (Figure 5B and Figure S4B). Additionally, we demonstrated that *Rbm14* has a role in the maintenance of genome integrity by regulating the alternative splicing of DDR‐related genes (Figure S4C). The altered splicing of these genes results in cell apoptosis and cell cycle arrest in both mouse embryos and ESCs, and thus inhibits gastrulation during early mouse embryogenesis.

Our previous work reported that knockout of *Rbm14* inhibited the expression of pluripotency‐associated genes such as *Oct4* and *Nanog* and thus influenced pluripotency maintenance of mESCs. RNA‐Seq also demonstrated decreased expression of genes associated with TGF‐beta and Wnt pathways.^23^ Interestingly, in this present study, we performed immunostaining and detected no substantial suppression of Oct4 and Nanog expression in *Rbm14* knockout mESCs, at least during the first 5‐7 passages. This contradiction might be due to the fact that distinct pathways are implicated in pluripotency maintenance of mESCs in different culture conditions. In our previous work, when mESCs were cultured in the serum + LIF system, the LIF/STAT3 signalling and the TGF‐beta signalling were essential for the pluripotency maintenance of mESCs.^40^, ^41^, ^42^, ^43^ While in this work, mESCs were maintained in the ground state in the 2i + LIF condition, in which a cocktail of two inhibitors for the MEK1/2 and GSK3 pathways (2i), has been proved to be sufficient to maintain mESCs with full pluripotency and LIF supplementation strengthens the robustness.^44^ In this condition, the TGF‐beta pathway has been shown to be less active.^45^

DNA damage is commonly associated with cell proliferation defects such as cell apoptosis and cell cycle arrest.^31^, ^32^ A previous study has shown that the cells in embryonic region, but not the extraembryonic region, are hypersensitive to DNA damage factors during early mouse embryonic development. Low dose of ionizing irradiation between E6.5 and E7.5 with 0.5 Gy disrupts gastrulation and markedly enhances the apoptosis in the embryonic region. In addition, depletion of RAD51^46^ and many other DNA damage repair factors, such as NBS1,^47^ PARP1/PARP2^48^ and BCAS2,^49^ results in early embryonic lethality. This indicates that the maintenance of genome integrity is critical during early mouse embryogenesis. In this study, we demonstrated that *Rbm14* regulates DDR by modulating the expression of DDR‐related genes, such as *Hnrnpk*,* Mdm2*,* Syce2*,* Emsy* and *Fance* through alternative splicing. Aberrant splicing of these genes results in the accumulation of DNA damages in cells and subsequently leads to cell proliferation defects. However, *Rbm14* may also regulate the cell proliferation and embryonic development by directly regulating the alternative splicing of related genes. This is because we also detected alternative splicing of genes related to transcription regulation, cell proliferation and embryonic development in the *Rbm14* knockout mouse ESCs.

Recent studies have reported the involvement of *Rbm14* in NHEJ by recruiting XRCC4 and XLF through Ku80 in HeLa and glioblastoma cells.^21^, ^22^ In our work, however, we detected no interaction between RBM14 and Ku80 or other NHEJ repair factors (Figure S4B). This may be due to the species specificity of *Rbm14* as all these previous studies were conducted in human cells.

In summary, we identified a pivotal role of *Rbm14* in the regulation of alternative splicing of DDR‐related genes and established that *Rbm14* is required for the maintenance of genome integrity during early stage development of mouse embryos. Our study revealed a novel role of RBPs in the regulation of early embryogenesis in mice.

## CONFLICT OF INTEREST

The authors declare no conflict of interest.

## AUTHOR CONTRIBUTION

WL, QZ and YZ participated in study design; JL, CXW, GHF, LLZ, GLC and HS collected data; GHF, JQW and YZ involved in methodology; CXW and GHF involved in data analysis; JL, CXW and GHF involved in manuscript preparation.

## Data Availability

The RNA‐Seq data and PAR‐CLIP‐Seq data have been deposited in Genome Sequence Archive of Beijing Institute of Genomics, Chinese Academy of Sciences (http://gsa.big.ac.cn/). The accession number for the sequencing data reported in this paper is CRA001790.
