# Supplementary material for: Rbm14 maintains the integrity of genomic DNA during early mouse embryogenesis via mediating alternative splicing
Source: Cell Prolif. 2019 Dec 3;53(1):e12724. doi: 10.1111/cpr.12724 (PMC6985654; doi:10.1111/cpr.12724)
Supplement: Supplementary file 1 [file CPR-53-e12724-s001.docx]

**SUPPLEMENTARY INFORMATION**


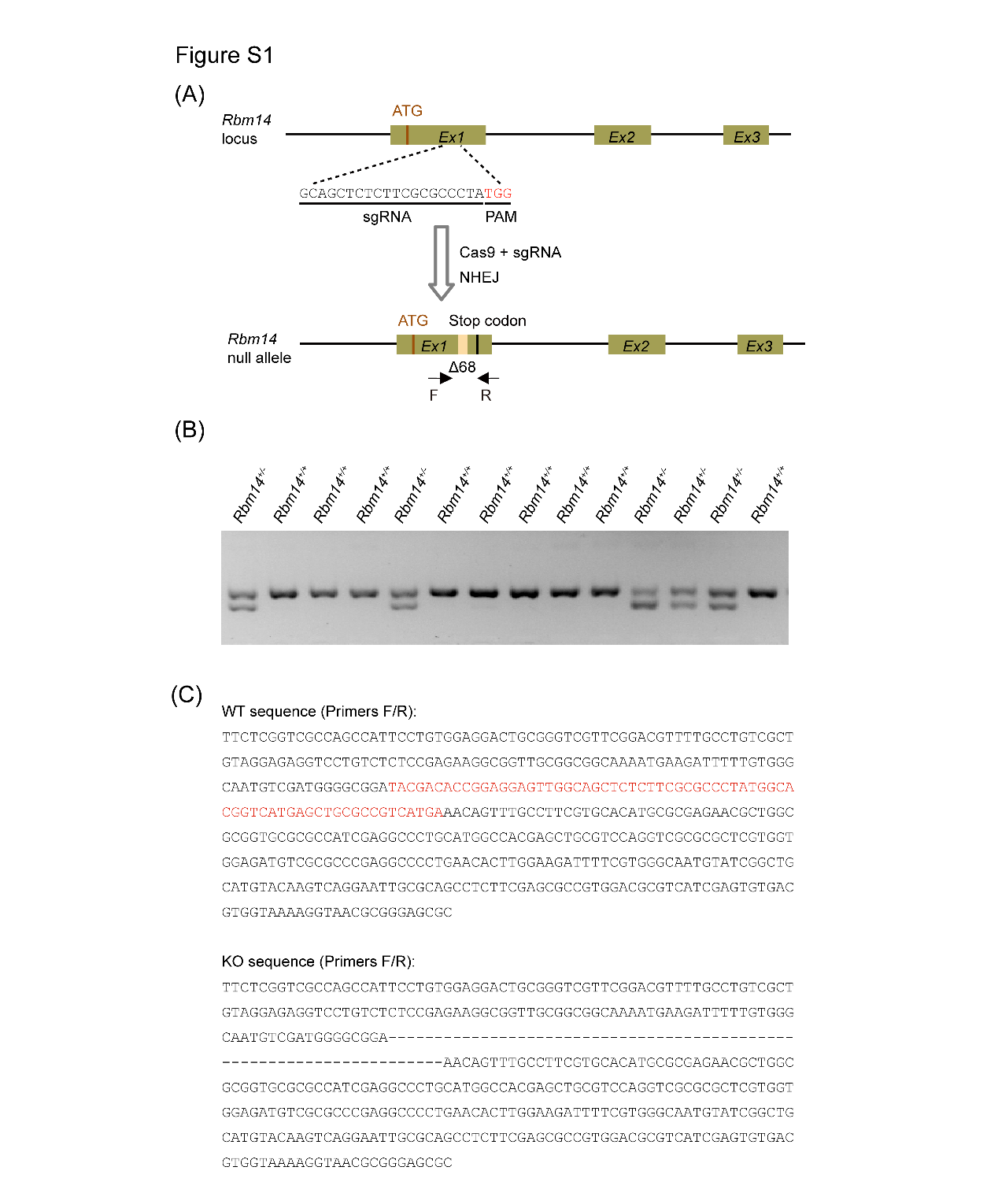


**Figure S1. Generation of the *Rbm14* knockout allele.** A, Schematic diagram showing experimental approaches to generate the *Rbm14* knockout allele in mice through clustered regularly interspaced short palindromic repeats (CRISPR)/Cas9-mediated gene targeting. The Cas9 mRNA and an sgRNA targeting the first exon of *Rbm14* were introduced into the fertilized eggs of ICR mice by microinjection. Cas9 along with the single guide RNA (sgRNA) cleaved DNA generated a double strand break (DSB) at the target site. A 68 bp deletion was generated at the DSB site as a by-product of DNA repair through NHEJ. The mutant allele causes frameshift and premature termination of translation at the N terminus of the protein. The mouse harboring this mutant allele is designated the Founder mouse. NHEJ, non-homologous end joining; Ex, Exon; F, forward primer; R, reverse primer. B, Genotyping of DNA extracted from tail tips confirmed heterozygous *Rbm14* knockout (*Rbm14^+/-^*) in F1 (denoting first generation) pups obtained after mating the male Founder mouse with the wild type (*Rbm14^+/+^*) female mice. C, Sanger sequencing of the target locus with primers F and R in both wild type and knockout allele. The sequence labeled in red shows the deleted region in the *Rbm14* knockout allele.

**
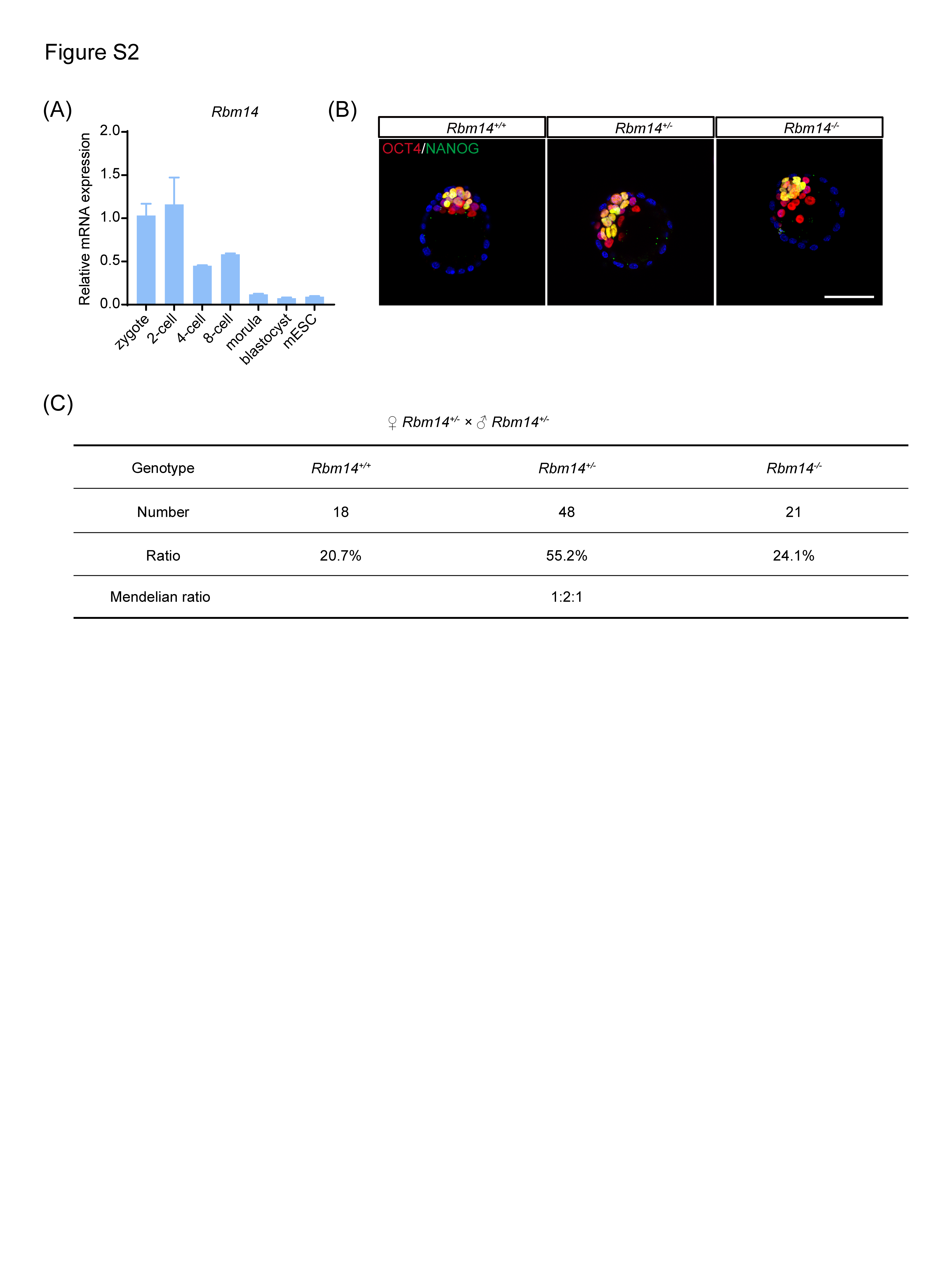
Figure S2. *Rbm14* knockout could support the development of mouse embryos from zygotes to blastocysts.** A, Q-PCR analysis of the expression pattern of *Rbm14* during pre-implantation development of mouse embryos. B, Representative immunofluorescent images of E4.0 embryos obtained after interbreeding of *Rbm14^+/-^* male and female mice for the early epiblast marker NANOG (green) and the inner cell mass (ICM) marker OCT4 (red). The nuclei were counterstained with 4′,6-diamidino-2-phenylindole (DAPI) in blue. Scale bar, 50 μm. The embryos were flushed from the uterus of the pregnant female mice at E4.0 sage. The embryos were stained and imaged and subjected to genotyping. At the late blastula stage, the inner cell mass (ICM) differentiates into two populations of cells: the NANOG^+^ epiblast proximal to the trophectoderm and the NANOG^-^ primitive endoderm facing the blastocoel. C, Quantification of these E4.0 embryos (*n* = 87) with different genotypes reveals no substantial influence on pre-implantation embryonic development upon knockout of *Rbm14*. The proportion of these embryos with different genotypes is highly in accordance with the theoretical Mendelian ratio.

**
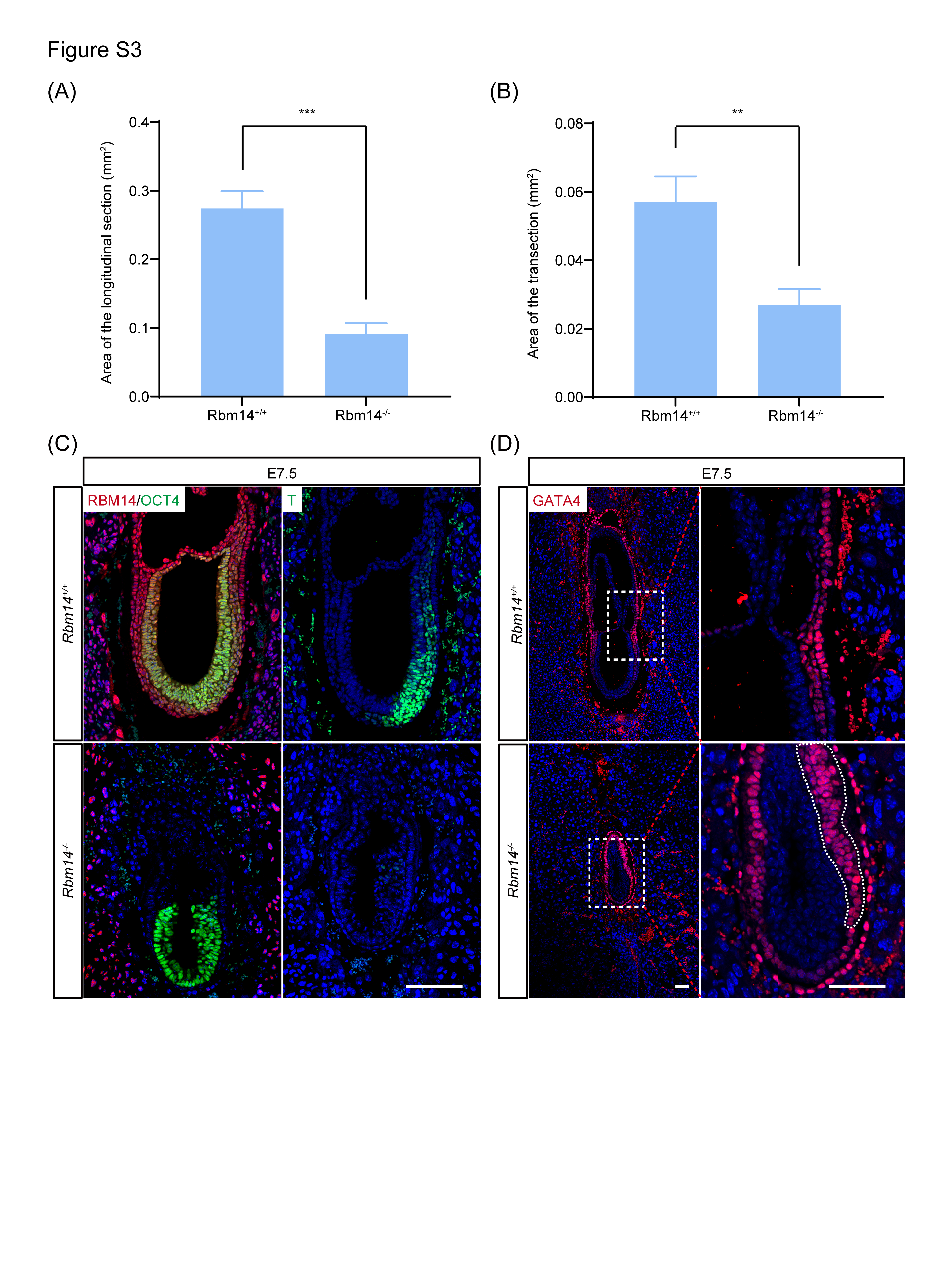
Figure S3. *Rbm14* knockout inhibits gastrulation during early mouse embryonic development.** A, The area of the longitudinal section of both the wild type and *Rbm14* knockout E7.5 embryos. B, The area of the transection of both the wild type and *Rbm14* knockout E7.5 embryos. Data are shown as mean ± s.e.m (*n* = 3). ***P* < 0.01, ****P* < 0.001, Student’s *t*-test. C, Representative immunofluorescent images of both wild type and *Rbm14* knockout embryos at E7.5 stage for RBM14 (red), OCT4 (green), and T (green). The nuclei were counterstained with 4′,6-diamidino-2-phenylindole (DAPI) and are shown in blue. Scale bar, 50 μm. Expression of the mesoderm *T* gene is suppressed in the knockout embryo. D, Representative immunofluorescent images of both wild type and *Rbm14* knockout embryos for GATA4 (red) at E7.5 stage shows accumulation of visceral endoderm cells in the *Rbm14* knockout embryo. The nuclei were counterstained with DAPI and are shown in blue. Scale bar, 50 μm. For either wild type or knockout embryos in these histological analyses, *n* = 3.

**
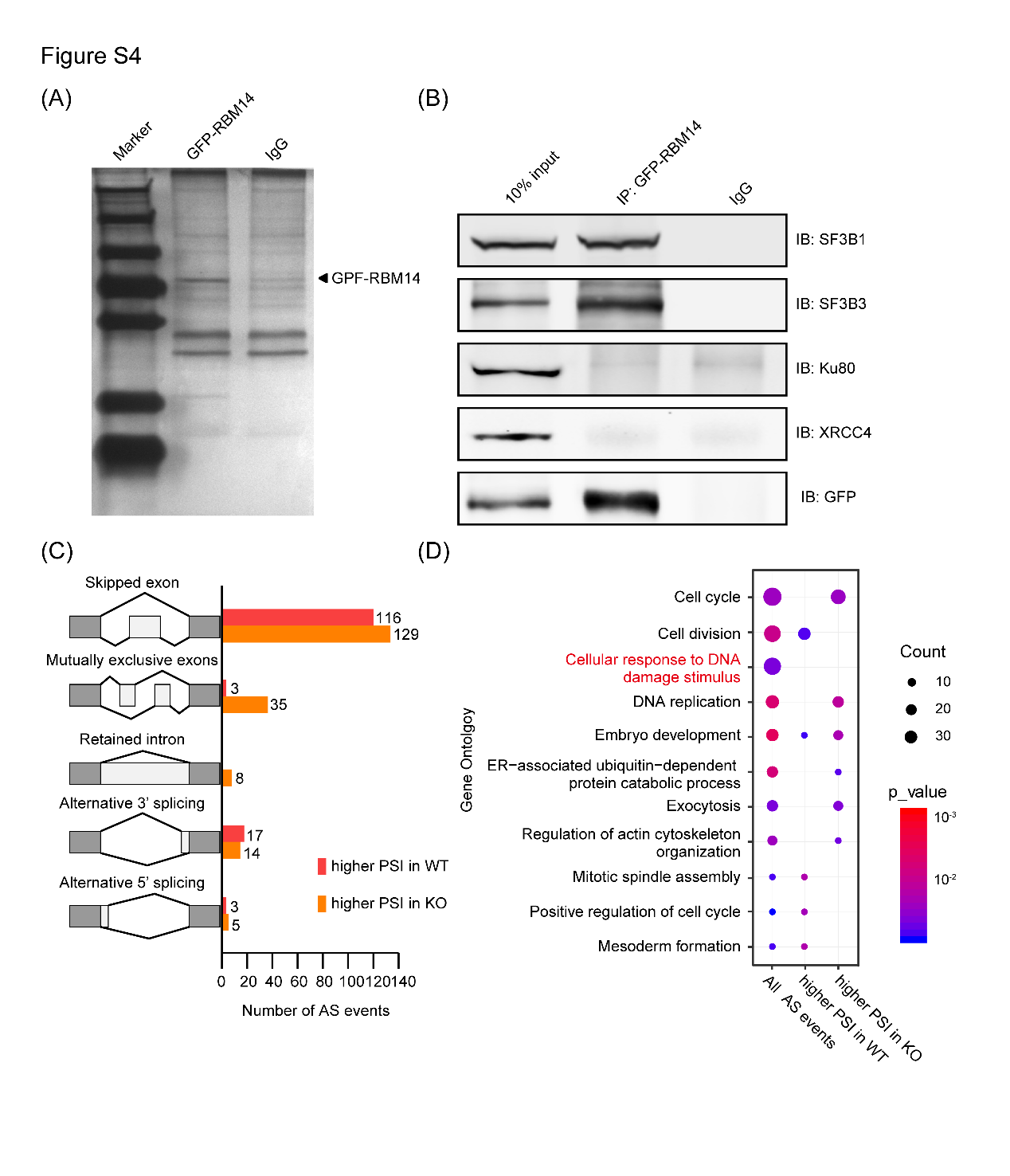
Figure S4. *Rbm14* regulates alternative splicing of DDR-related genes.** A, Representative image of silver staining for proteins immunoprecipitated with RBM14 using an anti-GFP antibody. The GFP-RBM14 band is indicated with a black triangle. Two independent biological repeats were used. B, Co-immunoprecipitation (Co-IP) reveals the interaction of RBM14 with splicing factors SF3B1 and SF3B3 but not non-homologous end joining (NHEJ)-related proteins Ku80 or XRCC4. C, The number of different alternatively spliced events between wild type and *Rbm14* knockout ES cells. And higher PSI in WT events are defined as the events with higher than 10% PSI in wild type ES cells compared to which in *Rbm14* knockout ES cells. And [vice](javascript:;) [versa](javascript:;) in *Rbm14* knockout ES cells. D, GO analysis shows that the alternatively spliced genes were enriched in “Cell cycle”, “Cell division” and “Cellular response to DNA damage stimulus” processes.

**Table S1. Sequences of the oligos and primers.**

**
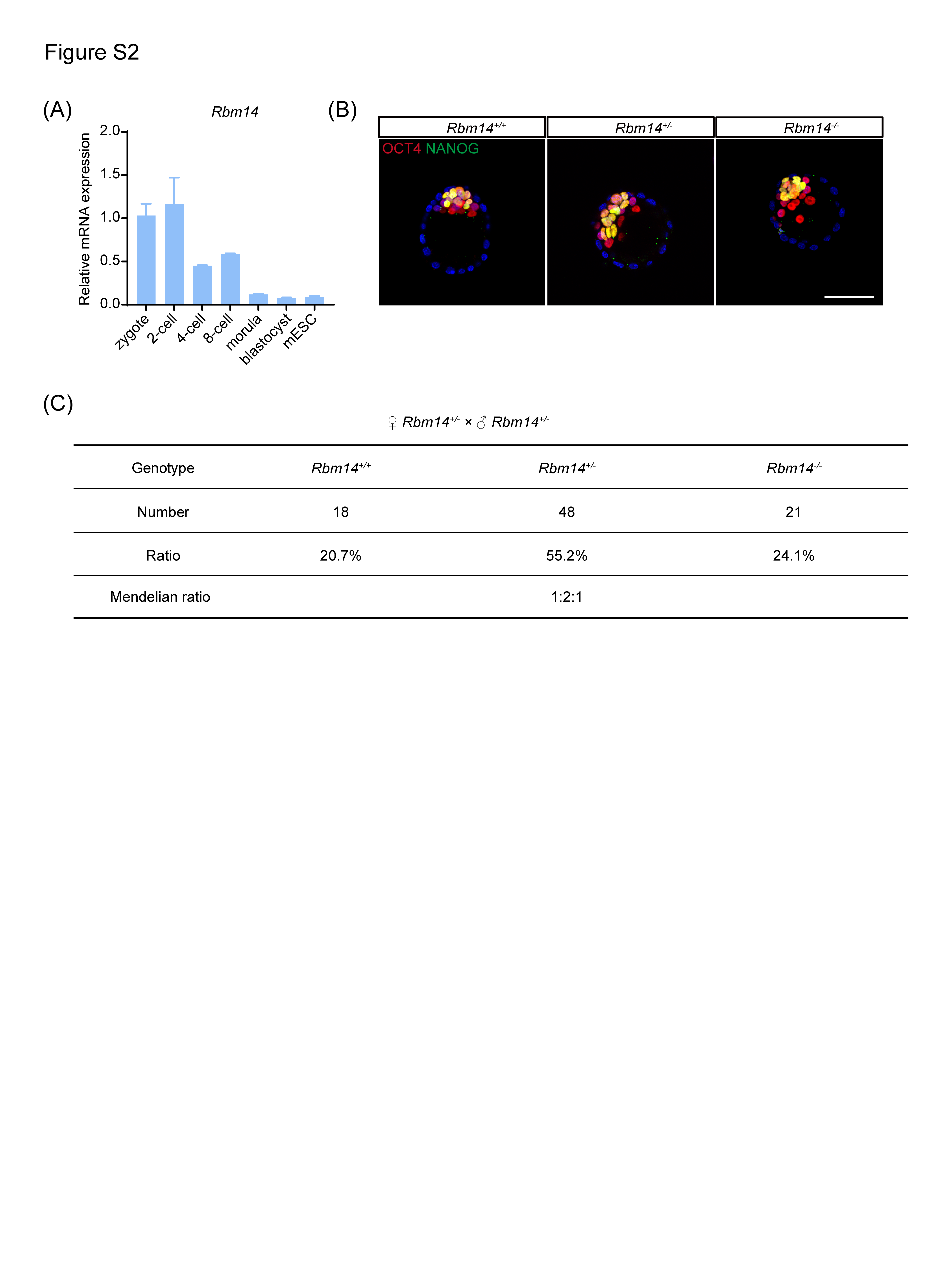
**

| Oligo/Primer | Sequence |
| --- | --- |
| U6-Rbm14-sgRNA-F | CCGGGCAGCTCTCTTCGCGCCCTA |
| U6-Rbm14-sgRNA-R | AAACTAGGGCGCGAAGAGAGCTGC |
| D-Rbm14-F | TTCTCGGTCGCCAGCCATTC |
| D-Rbm14-R | GCGCTCCCGCGTTACCTTTTA |
| Q-Rbm14-F | GTGGGCAATGTATCGGCTG |
| Q-Rbm14-R | CCCTTGGTTGAGAGTTCCACG |
| Q-GAPDH-F | AGGTCGGTGTGAACGGATTTG |
| Q-GAPDH-R | TGTAGACCATGTAGTTGAGGTCA |
| HNRNPK-AS-F | GACGCCATTATCCTCTGCTTCTC |
| HNRNPK-AS-R | TTCTGGCTGTTCGGTCTCCAT |
| Mdm2-AS-F | GGAGACCGACCGGACACC |
| Mdm2-AS-R | AACGGACTTTAACAACTTCAAAA |
| Syce2-AS-F | AGCATCGGCAGAGTGAGAACC |
| Syce2-AS-R | AAGCTCCATTTCCAGATGGTTG |
| Emsy-AS-F | AATCTACGTGCAACCCCAAACT |
| Emsy-AS-R | GCTGGAGTGACAAGGAATAGCATA |
| Fance-AS-F | CGTTCCTGGTGTTGCAGACG |
| Fance-AS-R | GCTGCTTGCAGGGCCTTCTT |

**Table S2. Primary and secondary antibodies used in the immunostaining and western blotting assays.**

| Antibody | Manufacturer | Catalog | Species | Dilution |
| --- | --- | --- | --- | --- |
| Rbm14 | Abcam | Ab70636 | rabbit | 1/2000 |
| α-Tubulin | Millipore | T6199 | rabbit | 1/1000 |
| Nanog | Abcam | Ab80892 | rabbit | 1/400 |
| Oct4 | Santa Cruz | Sc-9081 | goat | 1/500 |
| T | Santa Cruz | Sc-17743 | mouse | 1/500 |
| Eomes | Abcam | Ab23345 | rabbit | 1/400 |
| γH2AX | Merck | 05-636-I | rabbit | 1/500 |
| BrdU | Sigma | C8434 | mouse | 1/500 |
| Gata4 | Santa Cruz | Sc-1237 | mouse | 1/500 |
| Phospho-H3 (Ser10） | Cell signaling technology | 3377s | mouse | 1/1000 |
| 488-anti-mouse IgG | CiteAb | A21202 | donkey | 1/1000 |
| 647-anti-rabbit | Dianova | 711-497-003 | donkey | 1/1000 |
| cy3-anti-goat | CiteAb | A21432 | donkey | 1/1000 |
| cy3-anti-mouse | CiteAb | A10521 | goat | 1/1000 |
| 488-anti-rabbit | CiteAb | A11034 | goat | 1/1000 |
| 800CW anti-rabbit IgG | LI-COR | 925-32211 | goat | 1/10000 |
| 800CW anti-mouse IgG | LI-COR | 926-32210 | goat | 1/10000 |
